# Advance care planning and palliative medicine in advanced dementia: a literature review

**DOI:** 10.1192/pb.bp.114.046896

**Published:** 2015-04

**Authors:** Ketan Dipak Jethwa, Oluwademilade Onalaja

**Affiliations:** 1 University of Warwick and Coventry and Warwickshire Partnership NHS Trust; 2 Coventry and Warwickshire Partnership NHS Trust

## Abstract

**Aims and method** To assess the factors that affect the clinical use of advanced care planning and palliative care interventions in patients with dementia. A literature search of Medline, Embase and PsycINFO was performed to identify themes in advanced care planning and palliative care in dementia.

**Results** In total, 64 articles were found, including 12 reviews, and three key areas emerged: barriers to advanced care planning, raising awareness and fostering communication between professionals and patients, and disease-specific interventions.

**Clinical implications** Most of the studies analysed were carried out in the USA or Continental Europe. This narrative review aims to help guide future primary research, systematic reviews and service development in the UK.

Advance care planning is a targeted intervention that promotes autonomy for end-of-life decisions. It is particularly important in dementia where the illness impairs individuals’ decision-making abilities. Patients with advancing dementia experience significant comorbidities such as malnutrition and dehydration. They may have no advance care plan (ACP) in place and this can pose difficult management questions for their families and attending physicians concerning palliation and end-of-life care.

## Method

A search of Medline, Embase and PsycINFO was performed to investigate the current literature on ACP and palliative care in dementia. The following search protocol was used: ([‘advance care plan*’] AND [‘palliat*’ OR ‘palliative care’ OR ‘end of life’] AND [‘dementia’ OR ‘Alzheimer’s’]).

## Results

The search produced 64 journal articles, including 12 reviews. The titles and abstracts of these articles were reviewed. Those published in English and pertaining explicitly to palliative interventions or advance care planning in dementia were looked at more thoroughly.

The majority of the studies found were conducted in the USA or Continental Europe. This limits the generalisability of study findings to practice in the UK. For example, in the USA artificial nutrition is commonly used, whereas in the UK the National Institute for Health and Care Excellence (NICE) guidelines for dementia^1^ state that artificial nutrition should not commence if there is resistance to eating or difficulties in swallowing, related to progression of the disease. Differences in service configuration between countries also limit the generalisability of these studies, especially when considering the role of different agencies in delivering care. Assertive outreach in general psychiatry clearly demonstrates this. In the USA it is associated with improved outcomes and cost-effectiveness; however, these findings have not been replicated in Europe because of differences in the way services are organised.^2^

The purpose of this literature review was to identify key themes for future research and development and how they may relate to practice in the UK.

## Discussion

Our review of the relevant papers identified the following themes:

- barriers to advanced care planning
- raising awareness and fostering communication between families and health professionals
- management issues specific to patients with dementia.

### Barriers to advanced care planning

Patient-centred care involves the assessment of physical illness and its functional impact with consideration of the patient’s preferences, needs and values, whereas ACP is a tool that aims to enhance individual autonomy in addressing end-of-life care. This approach becomes challenging when patients lack capacity to make their own decisions. At present, relatively few patients have such care plans in place. The reasons for this are multifactorial and are likely to represent both patient- and health professional-specific barriers.

In a Spanish study, a cohort of patients with mild Alzheimer’s disease and ‘proven decision-making capacity’ were supported by a multidisciplinary team (MDT) to draw up an ACP.^3^ Of the 15 patients recruited only 1 drafted a plan. Psychiatric comorbidities (depression and anosognosia), complex family dynamics and a reluctance to engage with the process were cited as patient factors contributing to poor uptake. This initial study highlights the importance of identifying and managing psychiatric disorders in patients with cognitive impairment. This is especially important as improvement in affective symptoms may also result in some improvement in cognitive function, especially in the early stages of dementia.

Delivering advanced care planning and palliative care in acute general hospitals has also been investigated. An intervention, supporting carers, was delivered when a patient with severe dementia was admitted to hospital.^4^ A designated team discussed the various advanced care planning and palliative care options available and supported the formulation of an ACP. Thirty-three patients were recruited and even though discussions were well received only 7 eventually wrote an ACP. The poor uptake in this study may be related to the reluctance of carers to engage with the process because of the distress associated with the hospital admission of a relative. In addition, there is the possibility that once a patient has been admitted to hospital a carer may feel compelled to continue with management of the acute medical problem. In a cross-sectional survey of patients and their spouses it was found that there was moderate agreement between patients and their spouses when considering end-of-life care.^5^ However, if there was discord, spouses were more likely to ask for treatment. Despite this ambivalence, advanced care planning is important. Nursing models that emphasise advanced care planning, communication and comfort are associated with higher levels of satisfaction among family members when they are asked to reflect on their experience of the end-of-life care given to their relative.^6^

In this category, the following areas for service development and medical education were noted:

- identification and management of psychiatric comorbidity
- identification of appropriate settings for the delivery of ACP interventions
- exploration of the patients’ and families’ ideas, concerns and expectations.

### Raising awareness and fostering communication between families and health professionals

In a Dutch post-mortem chart review of 198 patients, advanced care planning and palliative care were discussed with 11% of patients.^7^ In this study, 62% of patients had palliative care records, 49% of cases were discussed at an MDT meeting and 76% of patients had a physician order limiting life-sustaining treatment. The current paucity of ACP and targeted palliative care interventions in dementia may relate to a poor understanding of the condition and its natural history. Dementia is a progressive and terminal disease. In a multi-site observational study, 94% of physicians thought of dementia as ‘a disease you can die from’ compared with 43% of families.^8^ However, in another study,^9^ 19% of the physicians questioned stated that they did not discuss ACP options with patients with mild to moderate Alzheimer’s disease. Of the 81% who did discuss advanced care planning, 47% addressed end-of-life care specifically. In families where dementia was understood as a terminal condition, patient comfort was rated more highly than in those where dementia was not viewed as terminal. This may reflect acceptance of the diagnosis and an understanding of the natural course of the condition, thus giving families the opportunity to prepare both materially and psychologically.

The physician plays a key role in ensuring patients receive appropriate palliation and end-of-life care. In a multicentre cross-sectional survey of 594 nursing homes in Belgium, it was found that patients were more likely to receive palliative care if they had input from a general practitioner (a doctor who may initiate palliative care).^10^

However, more often than not, these decisions are taken when patients are very dependent and have lost capacity.^11^ This may be following admission to an acute hospital. In a small survey of health professionals (*n* = 16), including physicians and specialist nurses, respondents reported feeling most confident in managing pain.^12^ There was, however, significant variation in the knowledge of opioid dosing, management of constipation and artificial nutrition in patients with advanced dementia. Given that these patients may not be able to articulate their discomfort, which may only manifest as increasing agitation, it is important that clinicians recognise and manage reversible causes of distress. The complexity of the illness and non-specific presentations mean the medical team may feel ill prepared to deal with specific end-of-life issues. This uneasiness is also present among nursing staff and can result in poor communication between staff and patients/carers.^13^ To tackle this, a role-play-based teaching package was delivered to palliative medicine fellows^14^ who afterwards felt more able to discuss ACP and identify caregiver burden. This type of teaching is effective but labour intensive. A 2-day residential course, the ‘Dementia Difference Workshop’, has been developed in Canada. At a focus group 1 year after the initial training session respondents reported feeling more confident in communicating with patients about ACP and that the course had led to a change in their practice.^15^ Internet-based e-learning is another alternative.^16^ However, although online courses are effective at delivering information, they may not directly help improve learners’ communication skills. Both role-play and internet-based teaching methods are used in UK medical education and provide a key opportunity for raising awareness.

Advanced care planning discussions are associated with an increased rate of plan formulation.^17^ It is important that such discussions are undertaken in a supportive manner/setting to ensure understanding and involvement. Patients’ educational level also seems to be an important factor, affecting understanding and uptake.^18^ The use of audiovisual media can help overcome this and make the material more readily accessible. Lack of communication and support are frequently cited as sources of stress for caregivers, especially when patients are admitted to nursing homes.^19^

It is important to clearly and accessibly document the capacity assessment, patients’ preferences and their proxy (if appropriate). This is important for medico-legal reasons, as patients’ wishes may also change. A retrospective chart review of 93 US patients^20^ enrolled in a ‘program of all-inclusive care for the elderly’ found that patients had on average two (range 0–4) documented discussions per year considering end-of-life issues. It was found that, after adjusting for the number of medical comorbidities, including dementia, the longer the patient was enrolled the less aggressive they wanted their medical care to be. At enrolment 34.4% of patients requested full medical treatment while shortly before death this figure was much lower at 6.5%. The enrolment period ranged from 1.0 to 6.4 years with 46% enrolled for more than 3 years. It is, however, unclear whether this change was related to perceived or actual deterioration in health, reduced quality of life or awareness of the natural history of dementia. The recommendations of the Nuffield Dementia Report 2009, which propose a form of proxy decision-making in collaboration with the family, have been suggested as an alternative to legally binding advance decisions which may not be flexible enough to allow for changes in patients’ preferences.^21^

Key areas for service development and medical education in this category were:

- educational interventions for doctors and other health professionals to raise awareness of ACP and palliative care in dementia
- improving access to advance care planning and palliative care information for patients
- standardising documentation and ensuring services are dynamic to follow changes in patients’ wishes.

### Management issues specific to patients with dementia

The timing and triggers for palliative intervention remain unclear. It may be instigated in a number of settings including nursing homes, hospices or acute hospitals. In a sample of 198 patients, identified in a post-mortem study, 54% had dementia and 95% experienced one or more ‘sentinel events’ before the initiation of palliative care.^7^ These included febrile illness, pain or behavioural disturbance.

In the UK, the majority of patients with dementia are admitted to hospital. Lack of clinical improvement or worsening clinical biochemistry are common indications for palliation. This may include discussions with families about ‘do not resuscitate’ (DNR) orders, the cessation of active treatment and initiation of symptom control. End-of-life care is initiated by senior physicians. In addition, junior doctors need to be supported in managing acute behavioural disturbance and general deterioration. There is scope to develop a targeted management framework that takes into account the benefits and side-effects of treatment.^22^

The acute hospital plays a key role in the palliative care delivered to patients. Hospital admissions are related to an excess of sentinel events in the community. The capacity of hospices and nursing homes to instigate supportive or palliative measures is currently limited. In the USA, a retrospective cohort study^23^ of 240 patients investigating ‘do not hospitalise orders’ (DNHOs) discovered that 83.8% of patients had a DNHO in place and 24.6% of patients had a hospital transfer in the 6 months preceding death. Factors found to be independently associated with DNHO were: aged older than 92 years, nursing home stay of more than 2 years, eating problems and the surrogate decision maker not being the patient’s child. A qualitative study in the north-east of England, using semi-structured interviews and including representatives from community, hospital and ambulance services,^24^ found uncertainty among staff about whether current services could meet patients’ wishes. The main concerns highlighted included: responsibilities of different groups, aspects of ACP that are legally binding and inconsistencies between the forms used by different agencies. Clarification of roles, standardisation of documentation and shared care between primary and secondary care are organisational and legal issues that need to be addressed to facilitate continuity of care.

Intervention offered by special care units is a relatively under-researched area. A post-mortem review of the care of 422 nursing home residents (263 had dementia) by semi-structured interviews with care staff and 293 family caregivers found that patients with dementia had less shortness of breath, but required more physical restraint or sedative medication for behavioural disturbance.^25^ Patients in residential care had more skin ulcers, poorer hygiene, less use of restraint and higher use of emergency medical services. This study was performed in the USA and no difference was found between patients with or without dementia in terms of pain, ACP, life-prolonging interventions or hospice use.

A further longitudinal study of 323 patients in 22 nursing homes in Boston, USA was performed.^26^ It revealed that 43.7% of patients were cared for in a special care unit where they were more likely to receive treatment for dyspnoea, had fewer hospitalisations and were less likely to be fed via a nasogastric tube. Special care units are nursing homes where the structural design, training and activity programmes provide a supportive social environment for patients with dementia. Patients in standard nursing homes were more likely to receive analgesia, had fewer pressure ulcers, and antipsychotics were less frequently used. Staff in special care units reported higher levels of satisfaction.^27^ Staff in special care units may have more experience in managing patients’ personal care needs and behaviours, whereas those in nursing homes may have more experience in assessing and managing pain and pressure sores. A probable confounder in this study is the possibility that patients with more behavioural disturbance and higher care needs are more likely to be cared for in special units. The primary care physician may be in the best position to recognise when referral to a hospice or specialist unit is required.^28^

Key areas for service development and medical education in this category are:

- integration of dementia and pre-existing palliative care services
- integration of dementia and general medical services
- identification and management of causes of behavioural disturbance in dementia
- facilitating transfer of information and patient records between primary and secondary care
- legal issues surrounding the use of ACPs.

### Advanced care planning and palliative care in UK dementia services

The 2008 *End of Life Care Strategy* published by the UK Department of Health was the first comprehensive strategy for dying people.^29^ There have subsequently been a number of initiatives to improve advanced care planning and end-of-life care for patients with dementia, cancer and other chronic conditions.

The National Council for Palliative Care (www.ncpc.org.uk) has a section charged specifically with improving the provision of end-of-life care in dementia. In particular they are working on strengthening ties with pre-existing dementia services and palliative care services to ensure access and coordination between services. In some areas they are working closely with Admiral Nurses (www.dementiauk.org/what-we-do/admiral-nurses), specialist mental health nurses with additional training in dementia care. Given the unpredictability of the illness and questions over the timing and triggers for palliative intervention, integration of these two services will be invaluable in ensuring ACPs are drawn up and that appropriate end-of-life care is available when patients require it.

In 2012, the prime minister announced that dementia is now a national priority.^30^ A Challenge on Dementia scheme was set up to raise awareness and improve services. One of its key aims is advanced care planning. The scheme highlights the following as examples of good practice that should be replicated across the country: a community-based approach using dementia-friendly environmental design and non-invasive assistive technology to help people remain in the community, and a psychiatry and general practice intervention to facilitate end-of-life care in the community. At a time of financial constraint and increasing demand for already stretched accident and emergency services, these interventions have been welcomed as means of reducing costs.

The experience of dementia special care units can offer insights that may be transferable to general hospital or nursing home settings. Patients in special care units experience greater comfort, treatment for dyspnoea and are less likely to be fed by a nasogastric tube. Having ‘dementia wards’ in general hospitals staffed by physicians, psychiatrists and specially trained nursing staff will help ensure patients receive appropriate medical care and pastoral support. In the community, ensuring nurses have generic medical skills, such as setting up subcutaneous fluids, will reduce the requirement for hospital admissions and will increase the nurses’ experience and confidence in delivering complex palliative interventions.

### Limitations

The heterogeneity of study methodology, setting, reported outcome measures and small sample sizes reduce the generalisability of our findings. For example, end-of-life care discussions in out-patient and in-patient settings have different confounders, which will affect responses and outcomes. The majority of the studies are also retrospective or use post-mortem data that are open to recall bias and/or have incomplete/inconsistent data collection. More studies need to be undertaken in the UK, with larger sample sizes and standardised methods of reporting outcomes, to ensure applicability in the UK and comparison between studies.

This review has focused on the organisational factors associated with advanced care planning and palliative care in dementia. However, the disconnect between the willingness of carers and health professionals to discuss these issues,^31^ and the low levels of uptake and engagement reported in the studies reviewed, call for more exploration. This would require a wider review incorporating psychosocial literature exploring personal, cultural and other influences that shape people’s expectations towards death and end-of-life care. The role played by a lack of information, misperceptions about the course of the illness and the setting in which advanced care planning interventions are delivered have been cited in the studies reviewed as possible contributing factors.
